# Resting State Functional Connectivity in Patients with Chronic Hallucinations

**DOI:** 10.1371/journal.pone.0043516

**Published:** 2012-09-06

**Authors:** Iris E. Sommer, Mareike Clos, Anne Lotte Meijering, Kelly M. J. Diederen, Simon B. Eickhoff

**Affiliations:** 1 Psychiatry Department, University Medical Center Utrecht & Rudolf Magnus Institute for Neuroscience, Utrecht, The Netherlands; 2 Institute of Neurosciences and Medicine (INM-1, INM-22), Research Centre Jülich, Jülich, Germany; 3 Department of Psychiatry, Psychotherapy, and Psychosomatics, Medical School, RWTH Aachen University, Aachen, Germany; 4 Institute for Clinical Neuroscience and Medical Psychology, Heinrich-Heine University, Düsseldorf, Germany; Centre Hospitalier Universitaire Vaudois Lausanne - CHUV, UNIL, Switzerland

## Abstract

Auditory verbal hallucinations (AVH) are not only among the most common but also one of the most distressing symptoms of schizophrenia. Despite elaborate research, the underlying brain mechanisms are as yet elusive. Functional MRI studies have associated the experience of AVH with activation of bilateral language-related areas, in particular the right inferior frontal gyrus (rIFG) and the left superior temporal gyrus (lSTG). While these findings helped to understand the neural underpinnings of hearing voices, they provide little information about possible brain mechanisms that predispose a person to experience AVH, i.e. the *traits* to hallucinate. In this study, we compared resting state connectivity between 49 psychotic patients with chronic AVH and 49 matched controls using the rIFG and the lSTG as seed regions, to identify functional brain systems underlying the predisposition to hallucinate. The right parahippocampal gyrus showed increased connectivity with the rIFG in patients as compared to controls. Reduced connectivity with the rIFG in patients was found for the right dorsolateral prefrontal cortex. Reduced connectivity with the lSTG in patients was identified in the left frontal operculum as well as the parietal opercular area. Connectivity between the lSTG and the left hippocampus was also reduced in patients and showed a negative correlation with the severity of hallucinations. Concluding, we found aberrant connectivity between the seed regions and medial temporal lobe structures which have a prominent role in memory retrieval. Moreover, we found decreased connectivity between language-related areas, indicating aberrant integration in this system potentially including corollary discharge mechanisms.

## Introduction

Auditory verbal hallucinations (AVH) are among the most common and distressing symptoms of schizophrenia and also affect persons with affective psychosis and some (e.g., borderline and schizotypic) personality disorders. While AVH are thus a highly prevalent feature of psychiatric illnesses, their underlying brain mechanisms remain elusive. Models proposed for their pathophysiology include the misattribution of inner speech [1], [2], increased top-down processing [3], recollection of verbal memory fragments [4], [5] and sensory-based bottom-up deviations [6], [7]. Functional MRI studies demonstrated activation of inferior-frontal and temporo-parietal areas during the experience of AVH [5], [8], [9] together with deactivation of medial temporal lobe structures at the onset of AVH [5], [10]. However, these symptom-capture studies do not provide information about brain mechanisms predisposing a person to experience AVH, i.e. the *trait* to hallucinate. Insight into aberrations of neuronal networks underlying such vulnerability, in turn, may be particularly relevant for guiding the development and evaluation of treatment strategies.

It could be hypothesized that a predisposition to AVH may be instantiated by aberrant connectivity between inferior frontal and temporo-parietal areas leading to a misattribution of inner speech [1], [2]. If this were to be the case, we would expect reduced functional connectivity at rest between the inferior frontal and the temporo-parietal areas of both hemispheres, reflecting disturbances within the language network underlying aberrant processing of ambiguous bottom-up signals [11]. This hypothesized reduced couplings between inferior frontal and temporo-parietal areas may render a person vulnerable to hallucinate in the auditory verbal domain. At the structural level, deviations in the fiber-tracts connecting these areas have been demonstrated with Magnetic Transfer Imaging (MTI) in schizophrenia patients [12] and in non-psychotic individuals with frequent hallucinations [13]. However, it is currently unclear if these structural deficiencies also lead to functionally decreased connectivity.

Alternatively, deviant connectivity between speech-related areas and the medial temporal lobe could give rise to spontaneous verbal memory recollection [4], [5]. A predisposition for intrusive memory fragments may be reflected in anomalous functional connectivity between cortical association areas such as the frontal, temporal and parietal cortex and memory related structures such as the hippocampus and parahippocampal gyrus. Such increased vulnerability for intrusive memory fragments may predispose a person for hallucinations per se, as the hippocampal-parahippocampal complex is involved in the recollection of memories from several modalities. Increased vulnerability to memory intrusions could results from either increased connectivity between the cortical and the medial temporal structures, or decreased (inhibitory) connectivity, or a combination of both.

Either of these hypothesized dysconnectivities may become evident in the absence of external tasks, given that the latter may override the endogenous dynamics of AVH. Therefore, aberrations in functional coupling that may predispose a person to hearing voices may become evident by examining resting state connectivity data. Resting state fMRI connectivity measures low-frequency fluctuations in the cerebral haemodynamics (around 0.01–0.1 Hz). Recently, attention has been focused on the possible origins of these slow variations. Various investigations suggested that these signal variations are of neuronal origin and characterize the neuronal baseline activity of the human brain in the absence of externally stimulated neuronal activity.

Resting state connectivity can be assessed by using Regions of Interest (ROIs); areas that are thought to play a major role in the trait under investigation, or without the predefinition of ROIs as in an independent component analysis (ICA). In this paper, we used the first approach as there is good evidence for the essential contribution of certain well-defined areas in AVH. In contrast, the data-driven ICA approach may also pick up other differences in resting state between the groups that may not be related to hallucinations given that lack of focus on a specific system. In this study we aim to investigate the vulnerability to experience AVH in patients who have experienced AVH.

A recent meta-analysis indicated consistent activation of the right inferior frontal gyrus (rIFG) and the left superior temporal gyrus (lSTG) during the experience of AVH [14], these regions therefore provide a strong model to assess dysfunctional connectivity underlying AVH. In the present study we thus compare resting state connectivity of these regions between 49 psychotic patients with chronic AVH and a group of well-matched controls in order to unravel network aberrations underlying the predisposition to AVH. For practical reasons, we selected the Regions of Interest (ROIs) from a single large fMRI study [5], as meta-analytic studies provide rather large ROIs. As the study by Diederen et al. was by far the largest study included in the meta-analysis, results of the meta-analysis are largely in agreement with this study. We selected the two seeds that showed the largest areas of activation, namely the left superior temporal gyrus and the right inferior frontal gryus. These two seeds also have functional significance as the left superior temporal gyrus may be involved in the auditory component of AVH; i.e. the fact that voices are being heard aloud, while the right inferior frontal area may be the place where the words and sentences that are later perceived as voices could be generated [15].

## Materials and Methods 

### Participants

Forty-nine patients with psychosis, as diagnosed according to DSM-IV criteria by an independent psychiatrist using the “Comprehensive Assessment of Symptoms and History (CASH)” [16], who all experienced AVH with a frequency of at least several times a day during at least one year were included in the study (cf. Table 1 for clinical and demographic details). Psychopathology was assessed at the day of scanning with the Positive and Negative Syndrome Scale [17]. For comparison, 49 healthy (as confirmed by the CASH interview) individuals matched for age, sex and handedness were also included. After complete description of the study to the subjects, the participants provided written informed consent into the study, which was approved by the Humans Ethics Committee of the University Medical Center Utrecht.

**Table 1:** Demographic and clinical description of participants.

Group	Patients, n = 49		Control subjects, n = 49
Age	38.5 years ±11.72		39.5 years ±14.9
Sex	20 males, 29 females		18 males, 31 females
Handedness	41 right, 8 non-right		41 right, 7 non-right
Mean time with AVH	13.9±12.2 years		No AVH
Diagnosis	43 Schizophrenia, 4 Schizoaffective Disorder, 1 Schizophreniform Disorder, 1 Psychosis NOS		No psychiatric diagnosis
Antipsychotic Medication	10 clozapine, mean dose 464 mg, 3 flufenazine, mean dose 30 mg, 7 risperidon, mean dose 3.6 mg, 8 olanzapine, mean dose 14.2 mg, 7 quetiapine, mean dose 514 mg, 1 penfluridol 10 mg, 5 haloperidol, mean dose 4 mg, 1 aripriprazol, 15 mg, 7 medication-free		all medication-free
Mean	Positive scale	16.2±3.7,	
PANSS scores	Negative scale	16.3±5.2,	
	Total	63±13.3	

### Data acquisition and processing

Resting state scans of eight minutes duration (600 blood-oxygenation-level-dependent (BOLD) fMRI images) were obtained on a Philips Achieva 3 T MRI scanner using the following parameter: 40 (coronal) slices, TR/TE 21.75/32.4 ms, flip angle 10°, FOV 224×256×160, matrix 64×64×40, voxel-size 4 mm isotropic. (PRESTO scans typically have shorter TR than TE times, as the whole head is scanned with each volume in stead of the slab-wise read-out of EPI scans). This scan sequence achieves full brain coverage within 609 ms by combining a 3D-PRESTO pulse sequence with parallel imaging (SENSE) in two directions, using a commercial 8-channel SENSE headcoil [18]. Participants were instructed to lie in the scanner as still as possible with their eyes closed yet stay awake (which was confirmed by post-scan debriefing). After scanning, patients were asked whether or not they had experienced AVH during the resting state scan. Likewise, healthy subjects were asked for AVH, which were denied by all of them.

The resting state scans were first corrected for head movement by affine registration using a two pass procedure. The mean PRESTO image for each subject was then spatially normalized to the MNI single subject template [19] using the unified segmentation approach [20] and the ensuing deformation was applied to the individual PRESTO volumes. Finally, images were smoothed by a 5-mm FWHM Gaussian kernel. Regions of interest (ROIs) used as seeds for the functional connectivity analysis were based on two activation clusters reliably associated with the state of AVH obtained from Diederen et al. [5] (table 2.): the right inferior frontal gyrus, MNI coordinates: 44,16,12 and the left superior temporal gyrus, MNI coordinates −48, 0,0. We selected regions from this large fMRI study rather than using seeds from the meta-analysis [21] because the latter would have provided very large and unspecific clusters.

**Table 2:** Areas used for seed regions.

Lobe	Area	MNI Coordinates			T-value	Cluster size
R Frontal	Inferior Frontal gyrus	44	16	12	4.31	243
L Temporal	Superior Temporal gyrus	−48	0	0	3.46	40

T-values, cluster sizes and locations of local maxima of activation during AVH in 24 patients, taken from Diederen et al. 2011. Threshold set at p<0.05 whole-brain FDR corrected.

Voxel time courses were extracted for all voxels within a 5 mm sphere around the centre of the particular clusters that were located in the grey matter of the individual subject.

Variance explained by the following nuisance variables was removed from the time series to reduce spurious correlations [22]–[24]: i) motion parameters derived from image realignment and their first derivative; ii) mean grey, white matter and CSF signal intensity per time-point; iii) coherent signal drifts reflected by the first five PCA components on the entire whole-brain data. All nuisance variables entered the model as first and all but the PCA components also as second order terms, as previously described by Behzadi et al. [25] and shown by Chai et al. [26] to increase specificity and sensitivity of the analyses.. Data was then band pass filtered preserving frequencies between 0.01 and 0.08 Hz [27], [28]. The time course of each ROI was then expressed as the first eigenvariate of the processed time series of all voxels associated with that region. A recent study showed that this method is able to detect valid correlation and anti-correlations during rest, which are not an artefact of the preprocessing method, but may reflect valid biological signals [29]. Moreover, it should be emphasized, that the current analysis focussed on differences between the two groups of subjects, which should not be affected by potentially confounds induced by preprocessing steps, even if these were to be present, as long as they do not systematically vary in effect between groups.

### Data analysis

For each subject we computed linear (Pearson) correlation coefficients between the time series of the seeds and any other grey matter voxel, which were then transformed into Fisher's Z-scores. Group analysis was then performed on these by an analysis of variance (ANOVA) across subjects using appropriate non-sphericity correction. In the ANOVA, both the main effect of functional connectivity as well as the group-difference between patients and controls were modelled for each seed. Inference on this random-effects analysis was then sought using linear contrasts. First, main effects of connectivity (across both groups) were calculated for both seeds and a conjunction analysis was performed on these to reveal regions showing significant coupling. Subsequently, functional connectivity maps of rIFG and lSTG were assessed for significant differences between patients and controls using unilateral T-tests. These differences in connectivity were evaluated by testing for either significant *positive* coupling across both groups (main effect) in conjunction with a *reduced* coupling in patients or significant *negative* coupling across both groups (main effect) in conjunction with *enhanced* coupling in patients as compared to controls indicating enhanced and reduced coupling. For all analyses, results were regarded as significant if they passed p<0.05, cluster-level FDR-corrected for multiple comparisons. Anatomical localizations were obtained using the SPM Anatomy Toolbox [30].

### Statistical analyses

To test whether the observed differences between patients and controls were additionally related to psychopathology, we extracted individual functional connectivity between the seeds and the peak voxels of the clusters showing significant differences. We then performed a Spearman rank-correlation between these and the individual scores on the “Hallucinatory behaviour” (P3) PANSS item to test for significant (p<0.05) associations. In a second follow-up analysis we divided the patients into those who actually experienced hallucinations during the scan and those who did not. Subsequently, we computed a one-way ANOVA between the groups on the individual functional connectivity scores between the seed and the peak of a significant cluster.

## Results

All participants stayed awake during the resting state scan. Thirty-one out of the 49 patients reported the experience of hallucinations during the resting state scan. None of the controls experienced hallucinations during the scan.

The analysis of the main effects for both seeds across both groups is provided in Supplementary Material S1. When resting state connectivity was compared between the patients and the controls, the following differences emerged.

### Increased connectivity

**Figure 1:**
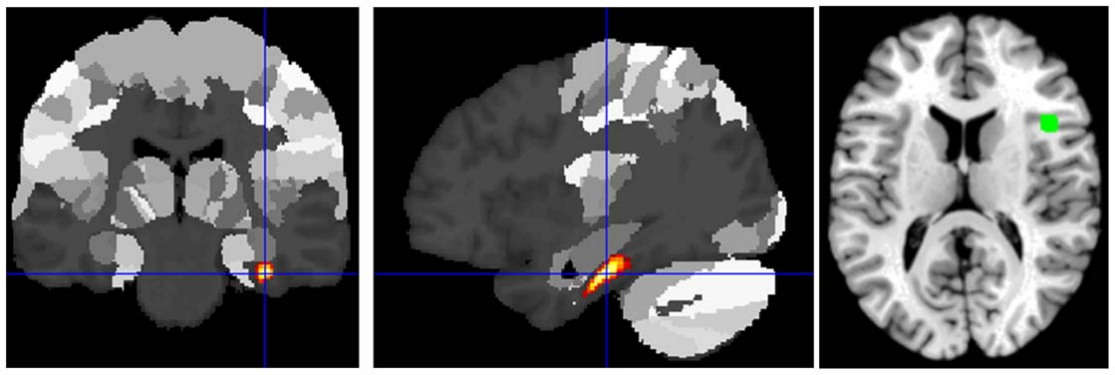
Increased connectivity with rIFG in patients as compared to controls. Increased connectivity with rIFG in patients as compared to controls was found in the right parahippocampal gyrus (p<0.05 cluster-level FDR-corrected).

The right parahippocampal gyrus showed significantly increased connectivity with the rIFG (Figure 1) in the patient group.

### Decreased connectivity

**Figure 2:**
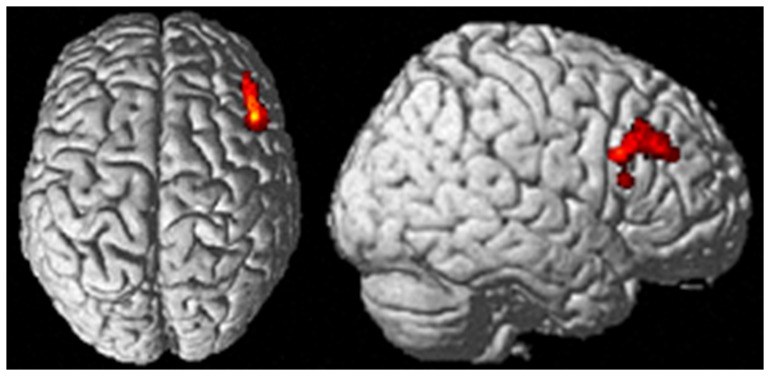
Reduced connectivity with rIFG in patients as compared to controls. Reduced connectivity with rIFG in patients as compared to controls was found in the right DLPFC/area 45 (p<0.05 cluster-level FDR-corrected).

Reduced connectivity with the rIFG in the patients was found for the right dorsolateral prefrontal cortex (DLPFC) just anterior to and slightly encroaching cytoarchitectonically defined Area 45 (Figure 2).

**Figure 3:**
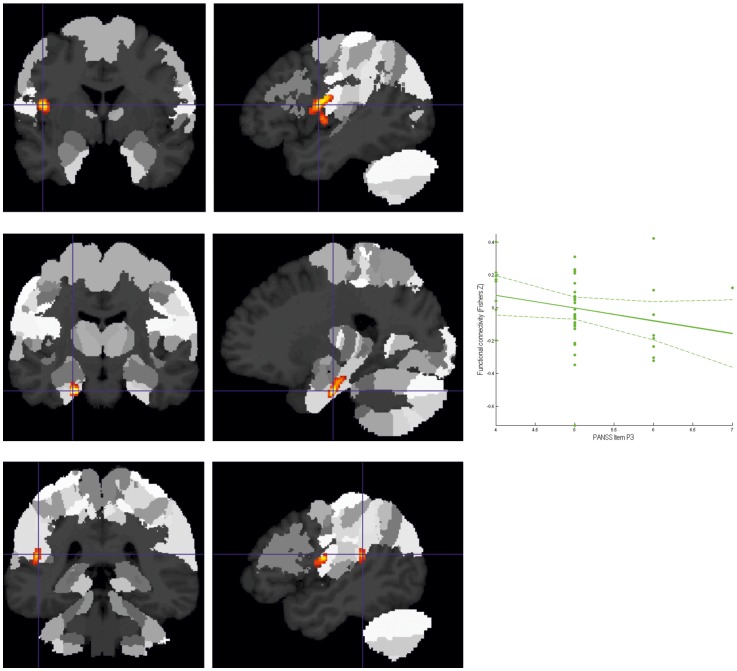
Decreased connectivity to lSTG in patients as compared to controls. **A.** Cluster of significantly (p<0.05 corrected) decreased connectivity with the lSTG in patients in the left frontal operculum/anterior insula shown on the MPM of the SPM Anatomy Toolbox. **B.** Cluster of significantly (p<0.05 corrected) decreased connectivity with the lSTG in patients in the left hippocampus (subiculum/entorhinal cortex) shown on the MPM of the SPM Anatomy Toolbox. **C.** Cluster of decreased connectivity (as a strong statistical trend) with the lSTG in patients in the left temporo-parietal operculum/retroinsular cortex.

Two regions showed significantly (whole-brain corrected) reduced connectivity to the lSTG in patients (Figure 3). The first was located on the left frontal operculum, extending into the anterior insula areas as well as the parietal opercular areas OP 3 and OP 4. The second was located in the left hippocampus, allocated predominantly to the subiculum and to a smaller extent the entorhinal cortex. Connectivity between the lSTG and the left hippocampus was not only reduced in patients but furthermore showed a significant negative correlation with the item P3 (hallucinations) of the PANSS rating scale.

**Figure 4:**
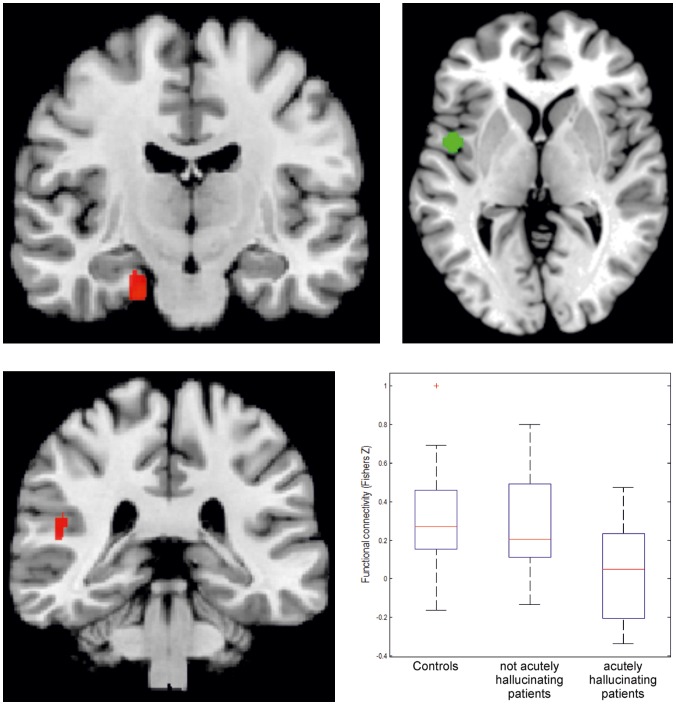
Differences in connectivity with lSTG, specified for patients who hallucinated during the scans and those who did not. **A.** Aberrations in functional connectivity between lSTG and the left entorhinal cortex gyrus differentiate between healthy controls. Separate findings are shown for patients showing the AVH trait and those acutely experiencing AHV (state). **B.** Aberrations in functional connectivity between lSTG and the left temporo-parietal operculum/retroinsular cortex differentiate between healthy controls. Separate findings are shown for the patients showing the AVH trait and those patients acutely experiencing AHV during the scan (state). The red crosses in these box plots represent data points outside 1.5 * IQR, i.e. outliers.

In order to assess state-effects of AVH on functional connectivity with our seed regions, we performed a follow-up analysis for those regions that showed disturbed connectivity in the overall group of trait carriers (i.e., the regions reported above) which assessed differences between controls, trait and state/trait-groups, i.e. individuals with acute AVH during scanning. For this purpose, we divided the patients into one group who hallucinated during the resting state scan, and those who did not. This analysis revealed that the reduction in connectivity between the lSTG and the left hippocampus was significantly stronger in those patients who hallucinated during scanning (Figure 4). That is, in these regions both trait (as demonstrated by the lower connectivity in non-acutely hallucinating patients compared to controls) and state-effects (as evidenced by the further reduction in the acutely hallucinating patients) were evident. For the remaining regions, including those showing abnormal connectivity with the rIFG, no differences were found between acutely hallucinating patients and those showing merely the trait to experience AVH.

## Discussion

We compared resting state functional connectivity of 49 psychotic patients experiencing chronic auditory verbal hallucinations (AVH) to 49 healthy controls. Analysis was seeded from the left superior temporal gyrus (lSTG) and the right inferior frontal gyrus (rIFG) as these areas are consistently activated during the state of AVH [5], [21] and are therefore expected to play a crucial role in AVH. In patients, the rIFG showed increased connectivity with the right parahippocampal gyrus and decreased connectivity with the right dorsolateral prefrontal cortex (DLPFC). Decreased connectivity with the lSTG in patients was revealed in the left frontal operculum and the left temporo-parietal operculum. Both opercular areas are considered part of the general neuronal frame work for language [28]. Furthermore, the lSTG showed decreased connectivity with the left hippocampus, this connectivity correlated significantly with the severity of hallucinations as measured in the PANSS interview.

To differentiate between trait and state aspects of AVH we divided the patient group into those who acutely hallucinated during scanning and those who did not. Here, only coupling of the lSTG with the left temporo-parietal operculum and hippocampus was more strongly decreased in the patients who actually hallucinated, indicating that these deviations may result from a combination of state and trait characteristics. Increased connectivity between the rIFG and the parahippocampus was equally strong in both patient groups.

### Dys-connectivity with the medial temporal lobe

Our analysis revealed disturbed interactions between both seeds and medial temporal lobe (MTL) structures, including increased connectivity between the rIFG and the right parahippocampal gyrus. This observation resonates strongly with a previous symptom capture study which revealed that cortical activations during AVH were preceded by significant signal changes in the parahippocampal gryus [5]. The MTL structures, especially the hippocampus [31], play important roles in both the consolidation and the retrieval of memories. This latter function may be involved in hallucinations, as hallucinations may be triggered by inappropriate recollections from memory. The parahippocampal gyri have a gatekeeper function as they integrate and transfer information from neocortical association areas to the hippocampus [32]. When this delicate interplay becomes deranged by aberrant connectivity, incidental information from memory may be recollected, giving rise to hallucinations [33]. The finding of both increased and decreased coupling between neocortical and MTL regions represents an important notion about the differentiated organization of the MTL and its interactions, challenging simple notions of “more” or “less integration”. Rather, deregulation of the usually well-balanced interactions between hippocampus, parahippocampal gyrus and neocortical association areas may represent a crucial pathomechanism for hallucinations. Since the hippocampus and parahippocampus regulate retrieval of memories in all modalities, we expect that deregulation between MTL and cortical association areas constitutes a predisposition to hallucinations in general and not specifically for hallucinations in the auditory verbal domain. Indeed, most of these patients who experience chronic AVH also experienced hallucinations in other modalities and can thus be expected prone to hallucinations in general.

### Dysconnectivity within the neocortical areas

Secondly, decreased connectivity in the patient group between the lSTG and the left frontal operculum was observed. We also found decreased connectivity between the rIFG and the right DLPFC. These findings may reflect decreased integration between areas mainly associated with the generation (rIFG) and interpretation (lSTG) of language, suggesting misattribution of inner speech as a potential pathomechanism. Furthermore, these regions are often found active during acute hallucinations [5], [21]. Mechanistically, such misattribution may be well reconciled with decreased connectivity by the notion of aberrant corollary discharge [34] failing to suppress the sensory consequences of self-generated actions. A possible mechanism would be that insufficient corollary discharge in the language system as reflected by decreased functional connectivity could result from microstructural alterations in the arcuate fasciculi, the most important fibre bundle between Broca's area and Wernicke's area [35] as observed by de Weijer and co-workers [12], [13]. Flaws or disintegration in the corollary discharge system of language networks may be specifically related to the attribution of internally generated speech to an external origin, which posed a predisposition specifically to hallucinate in the auditory verbal domain.

### Comparison to previous functional connectivity studies

Seeding from Wernicke's area (in close vicinity to our lSTG seed), Hoffman and colleagues [36] found increased connectivity in schizophrenia patients with a large subcortical cluster including the thalamus, midbrain and putamen, which is supported by the more localized findings of increased connectivity with the parahippocampal gyrus in the present analysis. Vercammen et al. [37] observed reduced functional connectivity between the left temporo-parietal junction and the right homologue of Broca's area in patients, while Gavrilescu et al. [38] demonstrated reduced connectivity of the bilateral auditory system. These studies thus point to decreased connectivity within the network of language-related areas in patients with AVH, a finding that was confirmed and extended in the present study. Rotarska-Jagiela et al. [39] found that the severity of positive symptoms correlated with functional connectivity of fronto-temporal and auditory networks in schizophrenia patients using an independent component analysis (ICA). In spite of the different approach taken in the present analysis, we could add evidence for the trait-specific aspect of this finding. We thus conclude that the present work is well in line with previous evidence but may provide more specific insights into disturbed circuits by seeding from areas shown to be engaged in AVH, separating state- and trait-dependent dysconnectivity and assessing the association between disturbed coupling and the severity of clinical symptoms.

### Limitations and technical considerations

A limitation of this study is the absence of a patient group without the trait to hallucinate. However, to our opinion, all patients with schizophrenia have a predisposition to hallucinate given their psychotic liability. The fact that a small proportion of schizophrenia patients have not experienced hallucinations may reflect adequate pharmacotherapy, rather than an absence of the trait.

The current approach of assessing functional connectivity of regions that have previously been implicated in the pathophysiology of hallucinations allowed for a model-based assessment of dysfunctional circuitry in patients suffering from AVH. This may represent a considerable advantage in terms of functional specificity as compared to purely data-driven approaches such as ICA, as ICA would also identify other differences between the groups unrelated to AVH. In turn, however, analyses based on a-priori information evidently have low sensitivity to aberrations in non-probed circuits.

The removal of noise from resting-state fMRI data as part of the preprocessing has been criticized because this process can introduce artificial anti-correlations [40], [41], [42]. However, component based methods such as PCA have been shown to be less susceptible to this effect while effectively reducing spurious correlations caused by noise [43], [44].

It must also be acknowledged, that the observed differences may not be absolutely specific to hallucinations, as patients and controls also differed in variables such as the presence of other psychotic symptoms, negative symptoms and medication status. While all these factors may have influenced connectivity patterns, the specific, localized aberrations of connectivity with areas activated during AVH should point to a pathophysiological contribution. This is in particular the case for those connections showing a significant state effect or an association with the severity of hallucinations as assessed with the PANSS rating.

In sum, we found two clear deviations in resting state connectivity in the patients with chronic AVH as compared to the controls. The first was aberrant connectivity between the seed regions that are active during AVH and MTL structures which have a prominent role in memory retrieval. The second deviation was decreased connectivity between language-related areas in the neocortex.

Aberrations between neocortical association areas and MTL structures may underlie a liability to experience hallucinations in general. In turn, reduced connectivity within the neocortical language network may indicate deficits in corollary discharge mechanisms as another potentially important pathophysiological mechanism for hallucination in the language domain specifically.

## Supporting Information

Supplementary Material S1 The analysis of the main effects for both seeds across both groups. The analysis of the main effects for both seeds across both groups demonstrated significant positive coupling between both seed regions with (in particular the left) inferior frontal lobes and anterior insula, dorso-lateral prefrontal cortex, inferior parietal lobules, right anterior temporal lobe and the posterior medial frontal cortex. In turn, both regions were significantly anti-correlated with anterior and posterior cingulated regions, the precuneus, right posterior inferior parietal lobule, bilateral inferior temporal lobe and right cerebellum. Positive coupling, i.e., functional connectivity, with the seeds is shown in green, negative coupling in red. Significantly decreased connectivity with the seeds in patients is superimposed on the main effect in cyan, significantly increased connectivity in patients is superimposed on the main effect in yellow below (all effects significant at p<0.05 cluster-level FDR-corrected).
(TIF)
