# Supplementary figures and images for: Resting State Functional Connectivity in Patients with Chronic Hallucinations
Source: PLoS One. 2012 Sep 6;7(9):e43516. doi: 10.1371/journal.pone.0043516 (PMC3435327; doi:10.1371/journal.pone.0043516)

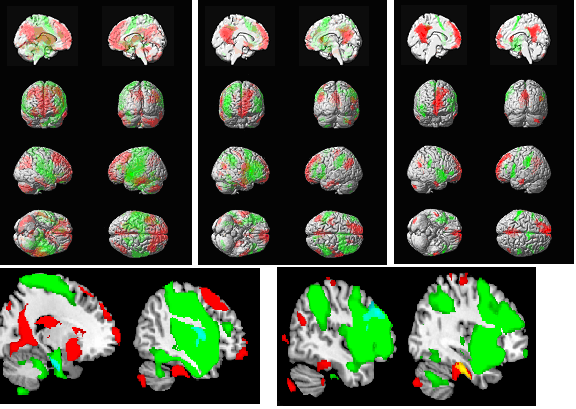

Supplement: Supplementary Material S1 — The analysis of the main effects for both seeds across both groups. The analysis of the main effects for both seeds across both groups demonstrated significant positive coupling between both seed regions with (in particular the left) inferior frontal lobes and anterior insula, dorso-lateral prefrontal cortex, inferior parietal lobules, right anterior temporal lobe and the posterior medial frontal cortex. In turn, both regions were significantly anti-correlated with anterior and posterior cingulated regions, the precuneus, right posterior inferior parietal lobule, bilateral inferior temporal lobe and right cerebellum. Positive coupling, i.e., functional connectivity, with the seeds is shown in green, negative coupling in red. Significantly decreased connectivity with the seeds in patients is superimposed on the main effect in cyan, significantly increased connectivity in patients is superimposed on the main effect in yellow below (all effects significant at p<0.05 cluster-level FDR-corrected). (TIF) [file pone.0043516.s001.tif]
